# In Vivo Human Apolipoprotein E Isoform Fractional Turnover Rates in the CNS

**DOI:** 10.1371/journal.pone.0038013

**Published:** 2012-06-04

**Authors:** Kristin R. Wildsmith, Jacob M. Basak, Bruce W. Patterson, Yuriy Pyatkivskyy, Jungsu Kim, Kevin E. Yarasheski, Jennifer X. Wang, Kwasi G. Mawuenyega, Hong Jiang, Maia Parsadanian, Hyejin Yoon, Tom Kasten, Wendy C. Sigurdson, Chengjie Xiong, Alison Goate, David M. Holtzman, Randall J. Bateman

**Affiliations:** 1 Department of Neurology, Washington University School of Medicine, Saint Louis, Missouri, United States of America; 2 Department of Medicine, Washington University School of Medicine, Saint Louis, Missouri, United States of America; 3 Department of Biostatistics, Washington University School of Medicine, Saint Louis, Missouri, United States of America; 4 Department of Psychiatry, Washington University School of Medicine, Saint Louis, Missouri, United States of America; 5 Department of Genetics, Washington University School of Medicine, Saint Louis, Missouri, United States of America; 6 Hope Center for Neurological Disorders, Washington University School of Medicine, Saint Louis, Missouri, United States of America; 7 Knight Alzheimer‘s Disease Research Center, Washington University School of Medicine, Saint Louis, Missouri, United States of America; University of Kentucky, United States of America

## Abstract

Apolipoprotein E (ApoE) is the strongest genetic risk factor for Alzheimer’s disease and has been implicated in the risk for other neurological disorders. The three common ApoE isoforms (ApoE2, E3, and E4) each differ by a single amino acid, with ApoE4 increasing and ApoE2 decreasing the risk of Alzheimer’s disease (AD). Both the isoform and amount of ApoE in the brain modulate AD pathology by altering the extent of amyloid beta (Aβ) peptide deposition. Therefore, quantifying ApoE isoform production and clearance rates may advance our understanding of the role of ApoE in health and disease. To measure the kinetics of ApoE in the central nervous system (CNS), we applied *in vivo* stable isotope labeling to quantify the fractional turnover rates of ApoE isoforms in 18 cognitively-normal adults and in ApoE3 and ApoE4 targeted-replacement mice. No isoform-specific differences in CNS ApoE3 and ApoE4 turnover rates were observed when measured in human CSF or mouse brain. However, CNS and peripheral ApoE isoform turnover rates differed substantially, which is consistent with previous reports and suggests that the pathways responsible for ApoE metabolism are different in the CNS and the periphery. We also demonstrate a slower turnover rate for CSF ApoE than that for amyloid beta, another molecule critically important in AD pathogenesis.

## Introduction

Apolipoprotein E (ApoE) is a 34 kDa protein which is highly expressed in the liver and the brain [1]. ApoE is a key regulator of lipid and cholesterol metabolism and transport. Humans have three different *APOE* alleles which result in isoforms of the ApoE protein differing by one or two amino acids: ApoE2 (cys112, cys158), ApoE3 (cys112, arg158), and ApoE4 (arg112, arg158). The prevalence of ε2, ε3, and ε4 alleles in European Americans is 7%, 78%, and 15%, respectively [2]. The amino acid substitutions affect the total charge and structure of ApoE [2], thereby affecting its binding to lipoprotein receptors and potentially the lipoprotein particle stability. ApoE found in the periphery and the central nervous system (CNS) are independent of each other and produced from different sources [3]. In the periphery, ApoE is produced predominantly by the liver and is preferentially found in VLDL [4]. In the CNS, ApoE is produced by astrocytes and microglia and is found in HDL-like particles. *APOE ε4* is currently the strongest genetic risk factor for developing Alzheimer’s disease (AD) [5]. Population studies have demonstrated that the *ε4* allele increases the risk of developing AD by either 3-fold (1 allele) or 12-fold (2 alleles) [6], resulting in an earlier age of onset of AD [7], [8]. Conversely, the *ε2* allele decreases the risk for developing AD [9]. In addition to AD, ApoE4 has been associated with increased risk for other neurological disorders including cerebral amyloid angiopathy, poor outcome after traumatic brain injury, and HIV-dementia [10]–[12]. The mechanism underlying the association between ApoE and AD may be related to differential effects of the ApoE isoforms on Aβ fibrillogenesis and clearance [5]. However, the mechanisms underlying the effect of ApoE4 on other neurological disorders, if they exist, are not known.

Peripheral blood ApoE metabolism is partially understood [13]–[17], where ApoE4 is catabolized twice as fast as ApoE3 [15]. However, little is currently known about ApoE turnover kinetics in the CNS. Due to a lack of *in vivo* studies, it is unclear whether a similar isoform-specific effect on ApoE turnover exists in the human CNS [18]–[21]. Furthermore, targeted replacement (TR) mice that possess the human ApoE isoforms substituted into the mouse gene locus have become popular tools for studying the effect of ApoE on the pathogenesis of neurologic diseases [5]. These mice express ApoE via the endogenous promoter, and as a result, the turnover of ApoE should reflect the natural synthesis and clearance rates of the protein [22]. In particular to AD, the ApoE TR mice have proven to be useful tools for studying the effect of the ApoE isoforms on both amyloid β (Aβ) deposition and clearance from the brain [23]–[26]. Despite considerable attention given to quantifying brain tissue and cerebrospinal fluid (CSF) ApoE concentrations [24], [27], [28], the kinetics of ApoE turnover in these mice have not been evaluated.

In this study, we quantified the *in vivo* kinetics of ApoE3 and ApoE4 in humans and human ApoE TR mice using stable isotope amino acid labeling coupled with mass spectrometry. For the human studies, peripheral venous blood and CSF were sampled during and after *in vivo* stable isotope labeling with ^13^C_6_-leucine (^13^C_6_-leu). The rate of appearance and disappearance of labeled ApoE isoforms in each compartment reflects their respective production and clearance rates. Utilizing an ApoE isoform-specific liquid chromatography/mass spectrometry (LC/MS) method [29], we compared ApoE isoform kinetics in both homozygous and heterozygous subjects in the periphery and CNS. For the mouse studies, we pulse-labeled several cohorts of mice with ^13^C_6_-leu and analyzed the *in vivo* kinetics of ApoE3 and ApoE4 by performing LC/MS analysis on brain homogenates.

## Results

### ApoE Isoforms in the Periphery have Different Turnover Rates

**Figure 1:**
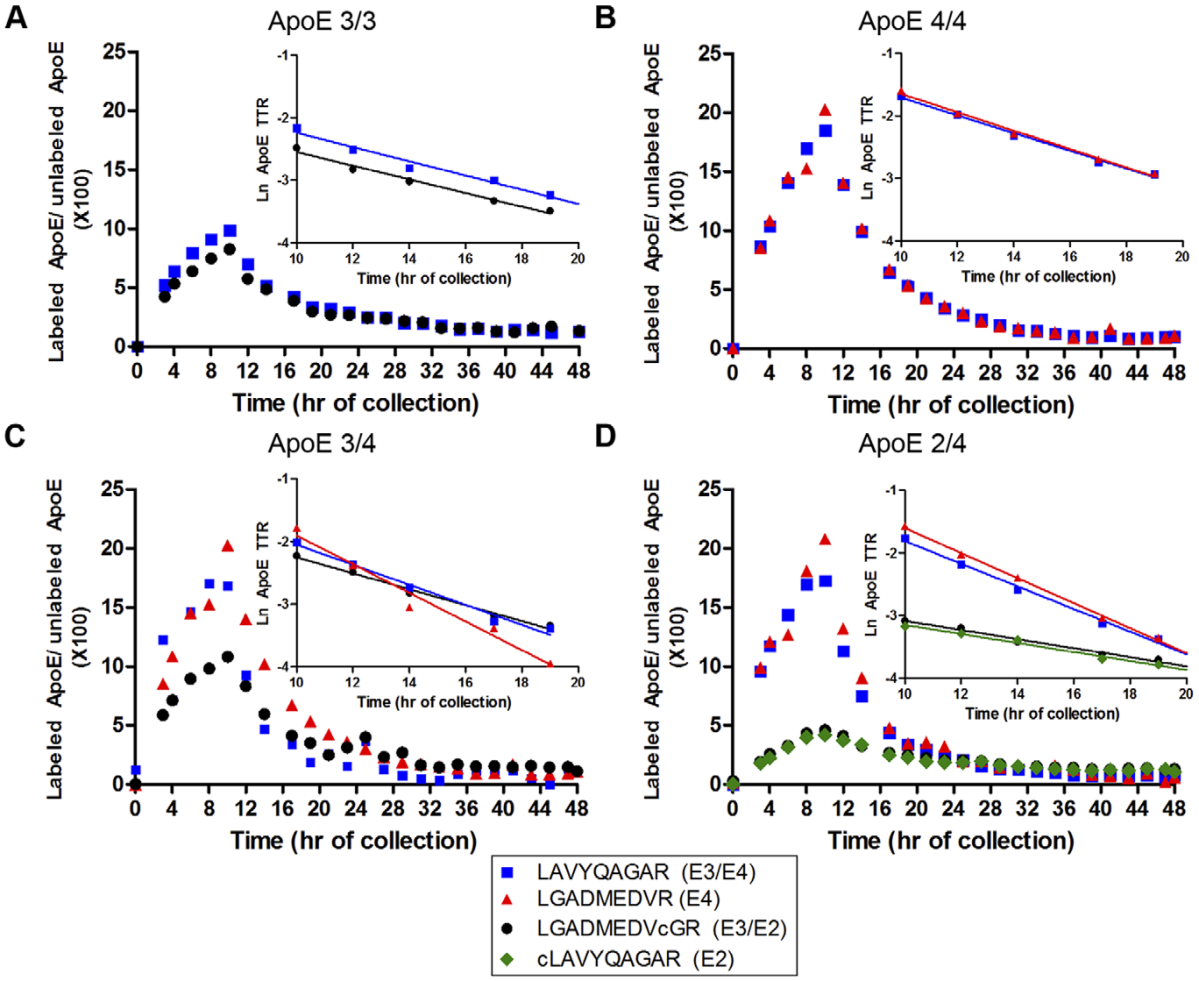
Plasma ApoE Isoforms have different turnover kinetics(ApoE4>ApoE3>ApoE2). ^13^C_6_-leu incorporation into plasma ApoE isoforms was analyzed from a representative individual for each genotype. The ^13^C_6_-leu incorporation peaked at 10 h with ApoE4’s maximum reaching 19.2%, ApoE3 9.9% and ApoE2 4.4%. The different isoforms have different clearance rates as indicated by the slope of the Ln plots (A–D insets). ***A–D*** **:** ***A*** *,* E3/3; ***B***, E4/4; ***C***, E3/4; ***D***, E2/4 (Blue: E3/E4 LAVYQAGAR, black: E3/E2 LGADMEDVcGR, red: E4 LGADMEDVR, green: E2 cLAVYQAGAR).

**Figure 2:**
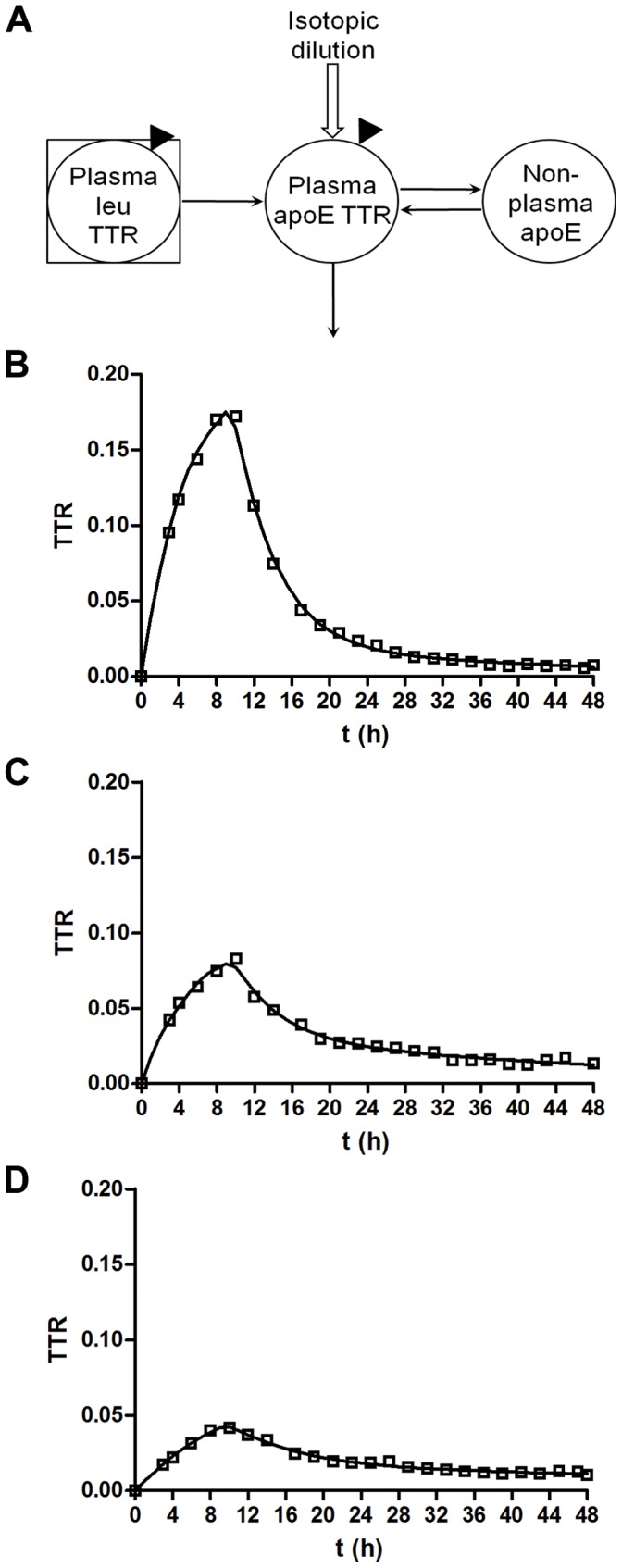
Representative compartmental model analyses of plasma ApoE. **A**. Peripheral ApoE compartmental model has 4 adjustable parameters: the plasma ApoE FTR, the rate constants for bi-directional exchange with the non-plasma space, and a scaling factor to account for isotopic dilution. **B**. ApoE4 peptide LAVYQAGAR from an ApoE2/4 subject. **C.** ApoE3 peptide LGADMEDVcGR from an ApoE3/3 subject. **D**, ApoE2 peptide cLAVYQAGAR from the same ApoE2/4 subject as in **B**. Solid line represents model fit to the data.

Studies have shown that ApoE isoforms have different kinetic properties in peripheral plasma [13]–[15]. In order to confirm that our ApoE isoform-specific LC/MS method yielded similar findings [29], plasma ApoE samples were analyzed from individuals labeled with ^13^C_6_-leu. CSF and blood were collected for 48 hours [30], [31]. ApoE labeling patterns obtained from an individual with each ApoE genotype are shown in Figure 1. The plasma ApoE TTR (tracer to tracee ratio) time course was characterized by a rapid rise during tracer infusion over 9 hours, followed by clearance of labeled ApoE. The TTR maximum at 10 h was 20% for ApoE4, 10% for ApoE3 and 4.5% for ApoE2 (Fig. 1) demonstrating that the isoforms have different turnover rates. The peripheral ApoE compartmental model (Fig. 2*A*) provided a strong fit to all sets of ApoE data (Fig. 2*B–D*). Kinetic parameters [fractional synthesis rate (FSR), monoexponential slope fractional clearance rate (FCR), and compartmental model fractional turnover rate (FTR)] of plasma ApoE are summarized in Table 1. Differences in ApoE isoform kinetics were found when each individual isoform was analyzed both between homozygous subjects and within heterozygous subjects. For example, ApoE4 protein turnover rates in homozygous subjects were approximately twice as fast the ApoE3 turnover rates in homozygous subjects (Fig. 1*A–B*, Table 1). Gregg *et al.* also observed in homozygotes that ApoE4 was catabolized 2-fold faster than ApoE3 [15]. Within the same subject, ApoE4 kinetic rates were double that of ApoE3 kinetic rates (Fig. 1*C*, Table 1). Furthermore, the LAVYQAGAR peptide, common to ApoE3 and ApoE4 proteins, exhibited kinetics that were intermediate between the plasma ApoE3 and ApoE4 peptides within ApoE3/4 heterozygotes (Table 1). ApoE2 had the slowest turnover rate. The plasma ApoE4 turnover rate was approximately 4-fold faster than plasma ApoE2 within ApoE2/4 heterozygotes (Table 1), which is similar to the 3-fold difference previously reported by Ikewaki *et al*. [13]. Therefore, consistent with previously reports [13]–[15], the plasma ApoE isoforms have different turnover rates, confirmed here using our isoform-specific LC/MS technique. ApoE protein levels in plasma also trended towards being lower in ApoE4 carriers (Table S1).

**Table 1:** Peripheral Plasma ApoE Kinetic Parameters.

Genotype	N	LGADMEDVcGR (E3/E2)	LAVYQAGAR (E3/E4)	LGADMEDVR (E4)	cLAVYQAGAR (E2)
		**FSR (h0–4) (%/h)**			
ApoE 3/3	1	6.9	8.3		
ApoE 3/4	1	8.2	8.2	9.0	
ApoE 4/4	1		12.3	12.7	
ApoE 2/4	1	2.7	14.3	14.6	2.7
		**Monoexponential slope FCR (h10–19) (%/h)**			
ApoE 3/3	1	10.5	11.4		
ApoE 3/4	1	12.9	18.2	22.9	
ApoE 4/4	1		15.2	15.7	
ApoE 2/4	1	7.4	19.6	20.6	7.2
		**Compartmental model FTR (%/h)**			
ApoE 3/3	1	11.4	14.0		
ApoE 3/4	1	15.1	18.2	35.2	
ApoE 4/4	1		16.4	16.2	
ApoE 2/4	1	5.7	23.1	20.2	4.5

### ApoE Isoforms Display Similar Kinetics in the CNS

**Figure 3:**
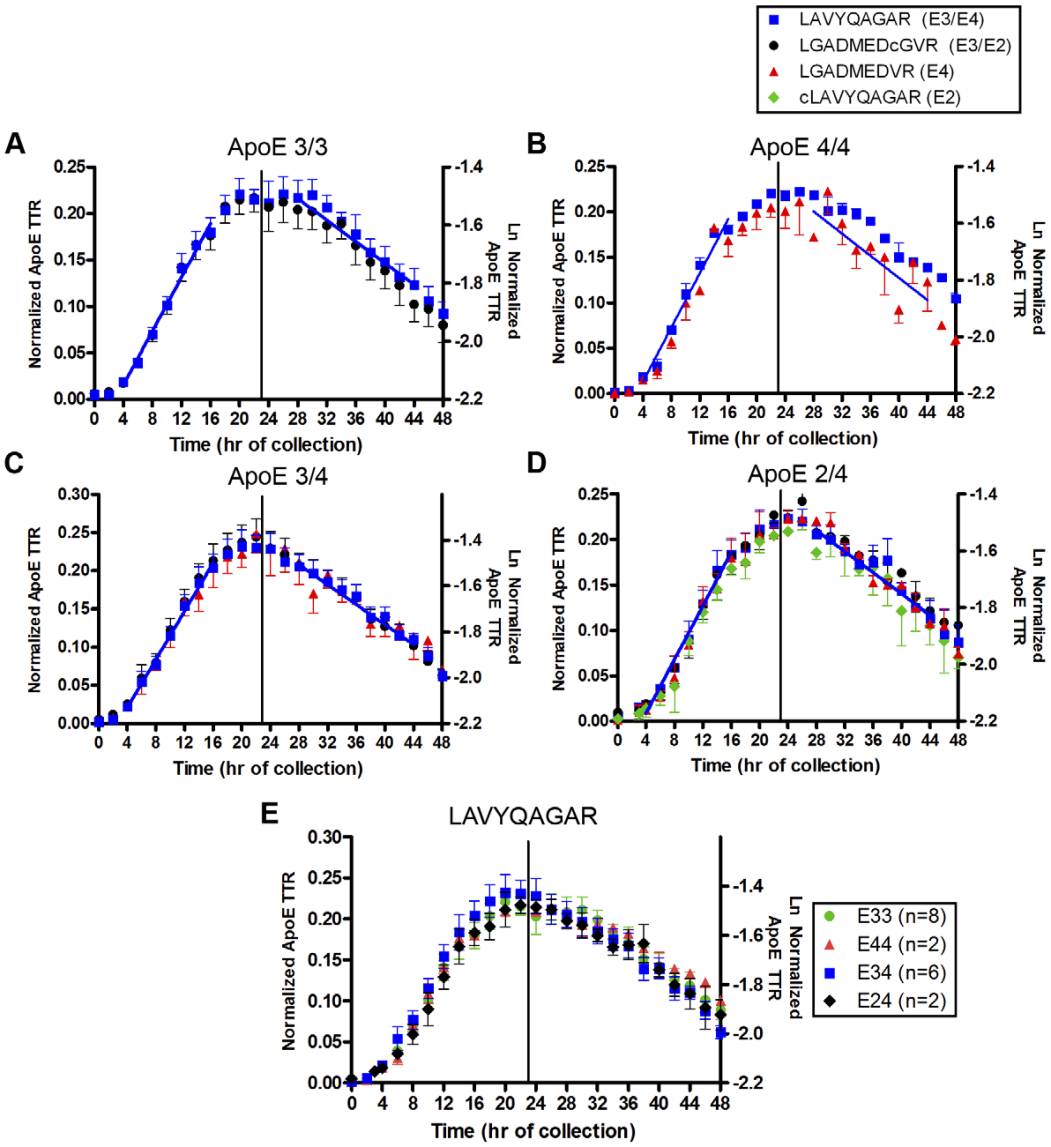
^13^C_6_-leucine labeling in CNS-ApoE isoforms in cognitively-normal young individuals. ^13^C_6_-leu incorporation into ApoE isoform-specific peptides was quantified by nanoLC/MS/MS. The ratios of the labeled to unlabeled ApoE were normalized to the plasma ^13^C_6_-leu precursor levels during the production phase (h0–22) to reduce inter-subject variability due to differential TTR of plasma leucine precursor. Individuals were grouped by genotype and their averages are shown in **A–D**: ***A*** *,* E3/3 (n = 8); ***B***, E4/4 (n = 2); ***C***, E3/4 (n = 6); ***D***, E2/4 (n = 2) (blue square: LAVYQAGAR, black circle: LGADMEDVcGR, red triangle: LGADMEDVR, green diamond: cLAVYQAGAR.). The linear regression of the means for h4–16 and h28–44 is shown for LAVYQAGAR to demonstrate the time points used for each individual’s FSR and monoexponential slope FCR calculations. **E.** The averages of the common peptide (LAVYQAGAR) for all four genotypes (n = 18) were compared (green circle: E3/3; red triangle: E4/4; blue square: E3/4; black diamond: E2/4). Error bars represent standard error of the mean (SEM).

To investigate whether a similar trend exists in the CNS, ApoE2, ApoE3, and ApoE4 kinetics were quantified in CSF using nanoLC/MS/MS and by monitoring isoform-specific ^13^C_6_-labeled and unlabeled tryptic peptides (E3/2: **L**GADMEDVc_112_GR, E4:**L**GADMEDVR_112_, E3/4: **L**AVYQAGAR, and E2: c_158_ **L**AVYQAGAR, **L** indicates site of ^13^C_6_ labeling, variable residues are denoted by subscript, and lower case ‘c’ indicates alkylated) [29]. The CNS ApoE TTR time course was characterized by a slow sigmoidal rise to a peak at ∼25 h (Fig. 3). The FSR and monoexponential slope FCR were calculated from the rising and falling portions of the TTR time course, respectively, for each CNS ApoE isoform-specific peptide (Fig. 3) and summarized in Table 2. With the exception of the E2/4 genotype (n = 2, FSR and monoexponential slope FCR ApoE2 vs. ApoE4 p<0.05, Table S2), there were no significant differences observed in ^13^C_6_-leu ApoE isoform peptide labeling, FSR, or FCR between genotypes. Consistent with this data, we also measured ApoE levels in the CSF and observed no statistical differences between genotypes (Table S1). The FSR and monoexponential slope FCR for the common peptide **L**AVYQAGAR were compared across genotypes (Fig. 3*E*). The average FSR for CNS-ApoE was 1.53±0.31%/h (n = 18, **L**AVYQAGAR) in this young, cognitively-normal cohort, and the monoexponential slope FCR was 2.0±0.42%/h (n = 18, **L**AVYQAGAR).

**Table 2:** CNS-ApoE Kinetic Parameters.

Genotype	N	LGADMEDVcGR (E3/E2)	LAVYQAGAR (E3/E4)	LGADMEDVR (E4)	cLAVYQAGAR (E2)
		**FSR (h4–16) (%/h)**			
ApoE 3/3	8	1.50±0.39	1.50±0.38		
ApoE 3/4	6	1.62±0.31	1.58±0.33	1.53±0.46	
ApoE 4/4	2		1.47±0.13	1.39±0.30	
ApoE 2/4	2	1.46±0.22	1.48±0.18	1.47±0.14	1.39±0.05
		**Monoexponential slope FCR (h28–44) (%/h)**			
ApoE 3/3	8	2.16±0.52	2.23±0.52		
ApoE 3/4	6	2.24±0.37	2.06±0.36	*2.78±0.92* *(n = 2* * *)*	
ApoE 4/4	2		1.6±0.11	*1.86(n = 1* * *)*	
ApoE 2/4	2	1.7±0.56	1.8±0.20	2.25±0.07	1.82±0.48
		**Compartmental model FTR (%/h)**			
ApoE 3/3	8	2.64±0.38	2.55±0.47		
ApoE 3/4	6	2.76±0.55	2.54±0.43	2.40±0.53	
ApoE 4/4	2		2.08±0.12	2.59±0.29	
ApoE 2/4	2	2.60±0.16	2.58±0.06	2.68±0.04	2.40±0.53

* Linear fits with r^2^<0.8 were excluded from mean calculations.

**Figure 4:**
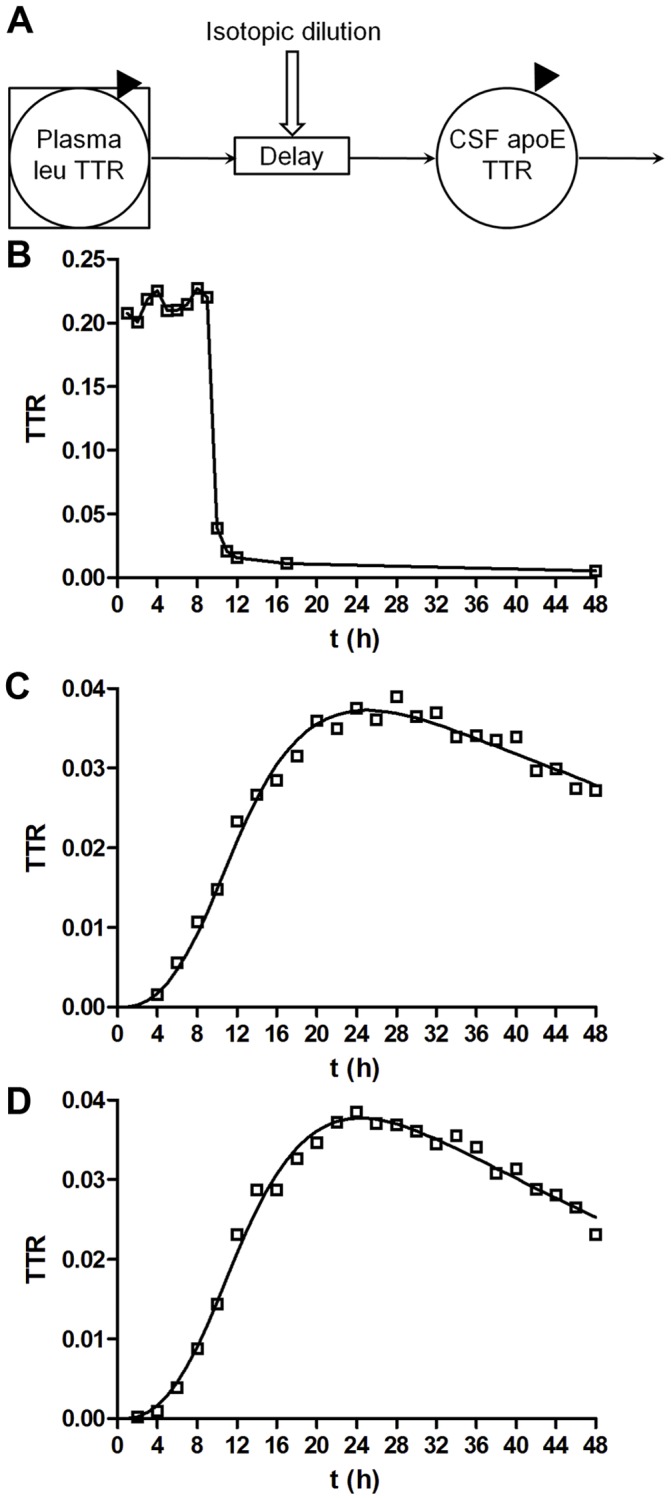
CNS kinetic modeling curves. **A**, CNS-ApoE compartmental model was used to describe whole-system CNS-ApoE turnover kinetics. The model is based on data from plasma leucine and CSF-ApoE TTRs (solid triangles). The plasma leucine TTR time course for a given subject is used as a “forcing function” to define the tracer availability for ApoE synthesis. The CNS-ApoE system comprises a delay element and a compartment that turns over, and accounts for isotopic dilution of the plasma leucine. The model has 3 adjustable parameters: the shape of the ApoE TTR time course is modified by adjusting the delay time and the rate constant for ApoE turnover, and the magnitude of the ApoE TTR is scaled by varying the degree of isotopic dilution. **B–D**, A typical compartmental model analysis from a single, representative, ApoE3/3 subject. **B**, Plasma leucine TTR remains elevated and does not return to baseline enrichment immediately after the tracer infusion is halted. **C–D**, The ApoE TTR time course exhibits a long time delay and sigmoid rise to a peak enrichment which is well described by the model. **C**, ApoE3 peptide LAVYQAGAR; **D**, ApoE3 peptide LGADMEDVcGR. Solid line represents model fit to the data.

The whole-system CNS-ApoE FTR was determined by fitting the full ApoE TTR time course to a compartmental model (Fig. 4*A*). Representative modeling curves are depicted in Fig. 4*B–D* and average kinetic rates for each genotype, grouped by peptide, are described in Table 2. The model provided a solid fit to the full ApoE TTR time course for all data sets (Fig. 4). No significant differences in kinetic parameters were observed between genotypes. In particular, there was no significant difference between ApoE isoform kinetics within either ApoE3/4 or ApoE2/4 heterozygotes. The whole-system FTR was 2.5±0.4%/h (n = 18, **L**AVYQAGAR). The monoexponential slope FCR was highly correlated (R^2^ = 0.71), and the FSR was less well correlated (R^2^ = 0.31), with the whole-system FTR.

### Human ApoE Kinetics in the Mouse Brain

**Figure 5:**
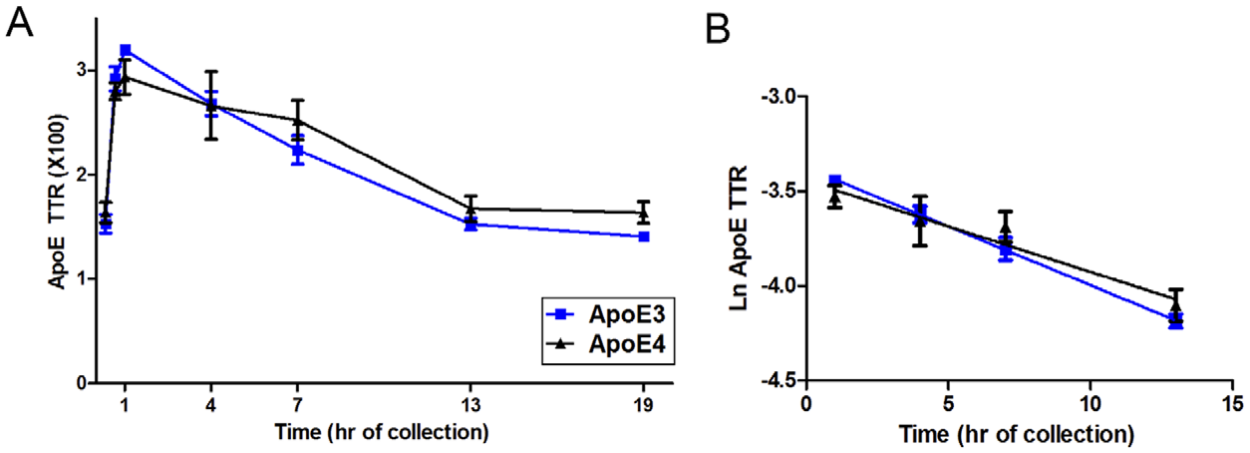
Brain ApoE kinetics in ApoE3 and ApoE4 targeted replacement mice. ApoE was extracted from brains of ApoE3/E3 and ApoE4/E4 mice labeled with ^13^C_6_-leucine. Similar kinetics were observed for ApoE3 and ApoE4 mice with monoexponential slopes of 6.2±0.48%/h and 4.8±1.12%/h, respectively (blue: ApoE3, black: ApoE4, n = 3–6 mice per time point, P  = 0.2817, error bars represent SEM).

Mice that have been genetically modified to express the human ApoE isoforms via the endogenous mouse ApoE promoter have become useful tools for studying the effect of human ApoE isoforms on various neurological conditions [5]. Several studies have analyzed ApoE levels in the brains of these mice, but the results have not been consistent [23], [24], [27], [28], [32]. In the current study, the brain kinetics of ApoE3 and ApoE4 were evaluated by pulse ^13^C_6_-leu labeling mice that were homozygous for either ApoE3 or ApoE4. The appearance of labeled ApoE in the mouse brain cortex was then quantified using LC/MS/MS. By plotting the ratio of labeled ApoE to unlabeled ApoE from the brains of different mice at various time points following the ^13^C_6_-leu pulse injection, a kinetic time course for ApoE3 and ApoE4 was obtained (Fig. 5A). ApoE turnover was characterized by a rapid rise in ^13^C_6_-leu labeling for the first hour following tracer injection, followed by a steady decrease in ^13^C_6_-leu labeling for the next 12 hrs. Similar to the analysis of the human data, the monoexponential slope FCR was calculated from the decreasing regions of the TTR time course for each ApoE isoform (Fig. 5B) (ApoE3 6.19±0.48%/h, ApoE4 4.80±1.12%/h, p = 0.2817). No statistically significant differences were observed between the FCR of ApoE3 and ApoE4. FSR values were not calculated because there were not a sufficient number of data points to accurately measure a linear front-end slope. We also measured brain ApoE levels in these mice by ELISA, and observed that ApoE4 mice had 12% less ApoE than ApoE3 mice (Table S1). Since the FCR values were not different between genotype, the small difference in protein levels is more likely due to changes in ApoE production. Therefore, we measured brain ApoE mRNA levels in the mice and found that ApoE4 mice have 20% lower mRNA levels than ApoE3 mice (Fig. S1).

### ApoE Turnover is Slower than Amyloid Beta Peptide Turnover in the CNS

**Figure 6:**
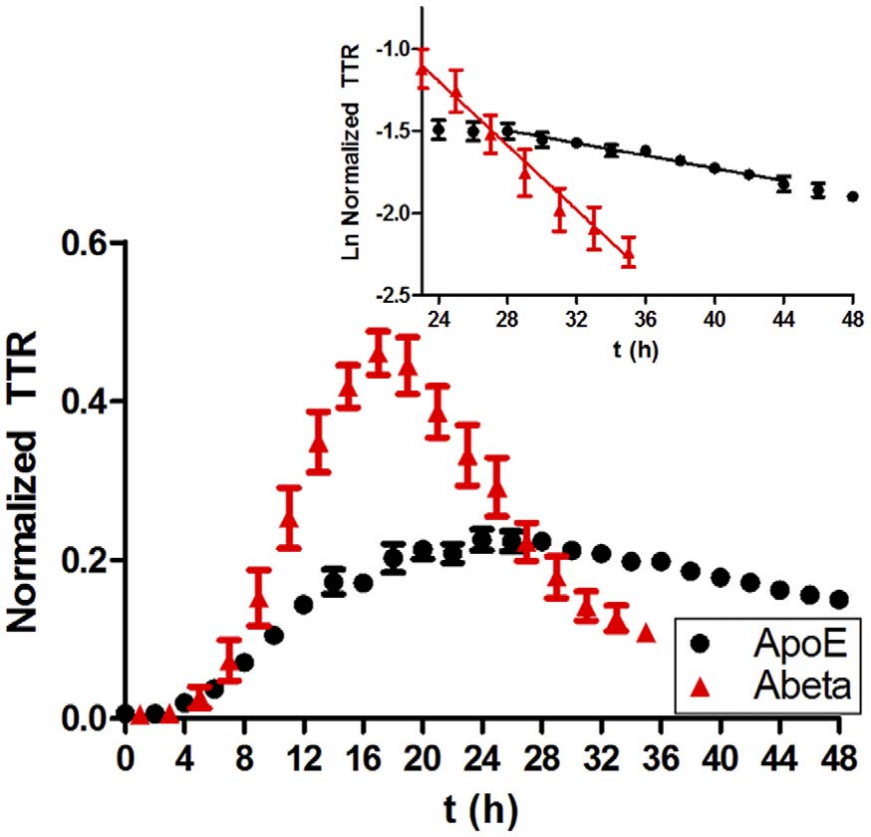
CNS-ApoE has slower kinetics than CNS-Aβ. The average of 4 YNC participants’ ApoE and Aβ (total) ^13^C_6_-leucine enrichment curves are shown. Aβ reaches a higher TTR than ApoE and clears the ^13^C_6_-leucine label 4× faster than ApoE. ApoE (total:LAVYQAGAR, black circle); Aβ (total, red triangles) (n = 4, error bars represent SEM).

It has been proposed that ApoE influences AD pathogenesis through regulating the clearance of Aβ in the brain, potentially through a direct interaction between Aβ and ApoE [5]. We were therefore interested in comparing the kinetics of ApoE and Aβ in the human brain. CSF TTR time courses for ApoE and Aβ were sequentially obtained from the same samples and compared during the human experiments (n = 4) (Fig. 6A). The Aβ kinetics for these 4 individuals were consistent with previous results observed in this population [31]. The maximal TTR for Aβ was twice maximal TTR for ApoE with Aβ reaching its maximum enrichment ∼8 hours prior to ApoE. The average monoexponential slope FCR for Aβ was 4.5 times greater than for ApoE (9%/h vs. 2%/h) (Fig. 6).

## Discussion

Using *in vivo* stable isotope labeling we report ApoE isoform kinetics in the CNS and peripheral circulation from young, cognitively-normal participants with different ApoE genotypes. In contrast to peripheral ApoE isoform kinetics, we found no significant differences in the CNS turnover rates for ApoE3 and ApoE4 in ApoE3/E4 heterozygotes (Fig. 3). We also observed no differences between ApoE3 and ApoE4 clearance rates in human ApoE TR mice (Fig. 5). We did find differences in CNS ApoE2 and ApoE4 FSR and monoexponential FCR (p<0.05, n = 2, Table S2) in ApoE2/E4 individuals; however, this trend was not reproduced in the FTR (compartmental modeling).

In humans, multiple studies have analyzed the effect of ApoE isoform status on ApoE protein levels in the CSF [19], [23], [33]–[35] and brain [20], [36]–[38]. However, results from these studies have not demonstrated any clear trend in ApoE levels, possibly due to issues with sample stability following collection, antibodies used for analysis, and heterogeneity in the subject population. Attention has also been devoted to measuring the ApoE levels in human ApoE TR mice. Since ApoE is expressed in these mice under the control of the endogenous mouse *APOE* gene promoter and regulatory elements [39], protein levels should be a reflection of the natural turnover rates of ApoE in the mouse CNS. Several studies have shown that mice with ApoE3 and ApoE4 have less total ApoE than mice with ApoE2 [23], [24], [28], [40], [41]. However, other studies have found no differences in the amount of ApoE between isoforms [27], [32]. The reason for these discrepancies is unclear, but may be due to the use of different ApoE antibodies, mouse genetic background or tissue lysis conditions.

Since the ApoE protein levels are intrinsically related to the turnover of ApoE, studying the kinetics of ApoE provides a mechanistic explanation for the differences, or lack thereof, in ApoE protein levels in the human CSF and mouse brain. Since we observed no differences in the FSR, monoexponential slope FCR, or FTR (compartmental model) of ApoE3 and ApoE4 in the CSF of humans, our data support the studies that describe no differences in brain ApoE levels between isoforms. However, the ability to accurately measure the levels of ApoE in the CNS remains an important issue that should be resolved. Many studies measuring the levels of the ApoE isoforms in the CNS rely upon techniques that use antibodies that might not recognize the isoforms with equal affinity. Therefore, future studies should attempt to measure ApoE levels in both the human and mouse brain using techniques that do not require antibodies, such as quantitative mass spectrometry.

Our plasma ApoE results are entirely consistent with previous reports of differential turnover rates for plasma ApoE isoforms (E4> E3> E2) [13]–[15]. These results indicate that ApoE turns over 3- to 6-fold slower in the CNS than the periphery. They also indicate that the similarity of ApoE isoform kinetics within the CNS is a result of physiologic and metabolic processes and not an artifact of our isoform-specific LC/MS method. We also observed a trend towards decreased levels of ApoE in plasma from E4 carriers (Table S1), consistent with previous results that observe highest ApoE levels in E2 carriers and lowest in E4 carriers [42], [43]. Since our labeling method confirmed the expected peripheral ApoE kinetic differences among individuals with different genotypes, we are confident that the CNS kinetic measures are valid and reflect physiologic and metabolic ApoE processing in the CNS.

Given the consistency of our plasma ApoE results with previous reports, the striking differences between CSF and plasma ApoE labeling time courses within all 4 individuals demonstrate that CNS-ApoE synthesis and clearance are largely independent of peripheral ApoE kinetics (compare Figs. 1 & 3). These results are consistent with previous findings showing that plasma and CSF ApoE concentrations are not correlated [44], and that brain and peripheral ApoE are generated in separate compartments [3], [45].

The FSR and monoexponential slope FCR kinetic parameters provide simple and convenient indices for ApoE turnover rate, but these approaches use only a subset of available data points and utilize assumptions that require validation. The compartmental models provide a more optimal description of whole-system CNS or peripheral ApoE kinetics because they provide excellent fits to the full ApoE TTR time course by incorporating the actual shape of the plasma ^13^C_6_-leu time course and incorporate features described by the physiology (a long time delay to represent CSF fluid transport, and a non-plasma exchange compartment for plasma ApoE). Compartmental models have been derived for plasma apolipoprotein kinetics [17], [46]–[48], but this is the first model that describes human CNS-ApoE kinetics.

The CNS ApoE turnover rate in young individuals is 1.5–2.5%/hour (half-life of ∼1 day), which is approximately 4-times slower than the turnover rate for the total CNS Aβ peptide (Fig. 6) [31], and approximately 10-times slower than CSF turnover [49]. The finding that fractional clearance rates differ for two different CSF proteins measured in the same person strongly supports the notion that protein kinetic rates measured in CSF reliably reflect CNS protein turnover and not just CSF turnover [50]. A slower CSF ApoE turnover rate compared to amyloid-beta may be expected given that ApoE recycles into and out of cells.

This stable isotope labeling approach for differentially quantifying protein isoform specific turnover rates may be applied to *in vitro* or *in vivo* model systems and used to inform about differences between protein isoform kinetics. No difference in CSF ApoE isoform-specific turnover rates was observed in this young, cognitively-normal cohort. However, the possibility remains that isoform-specific differences could emerge with age or during the onset and progression of disease. Future studies could use our labeling methodology to address these possibilities. This approach could also be used to evaluate the effects of therapeutics on ApoE production or clearance, such as LXR agonists or agents that modify ApoE receptor levels.

*In vivo* stable isotope labeling coupled to an isoform-specific LC/MS method enabled the first study of ApoE kinetics in the human CSF. Using a novel multi-compartmental model for CNS, we quantified CSF ApoE turnover rate to be ∼2%/h in young, cognitively-normal individuals. Our results suggest that there is no significant difference between the turnover of CNS ApoE3 and ApoE4 in young, cognitively-normal adults or between ApoE3 and ApoE4 TR mice. This is in sharp contrast to the ApoE isoform-specific differences in turnover rates observed in the peripheral venous blood. We also demonstrate that CNS Aβ turnover rate is roughly 4 times faster than ApoE.

## Materials and Methods

### Definitions of Samples and Kinetic Compartment Terminology

To provide a specific and consistent terminology with prior work, we refer to samples as brain, CSF, or plasma. The CNS *in vivo* labeling kinetics not only measure CSF kinetics, but also account for the site of production (astrocytes in the brain) and all clearance mechanisms in the brain, ISF, and CSF up to the point of sample collection [50]. Therefore, we refer to the labeling kinetic compartment as the CNS compartment. With peripheral kinetic measures, production and clearance are dependent on liver, blood, and interacting cells and molecules. Thus, we define and refer to kinetic compartments as either brain, CNS (including CSF), or peripheral compartments.

### ^13^C_6_-leucine-ApoE Standards

^13^C_6_-leucine-ApoE standards were collected from immortalized mouse astrocytes derived from ApoE knock-in mice expressing human ApoE2 or ApoE4 [51]. Cells were grown to near confluency in 10% FBS, 0.2 mg/mL geneticin (Invitrogen, Carlsbad, CA), 1 mM sodium pyruvate, and DMEM; then the media were changed to serum-free media: DMEM/Ham’s F-12 containing 1% N-2 supplement (Invitrogen), 1 mM sodium pyruvate, 3 µM 25-hydroxy-cholesterol (Sigma-Aldrich, St. Louis, MO), and 0–20% ^13^C_6_-leucine (^13^C_6_-leu) (105 mg/L, 98% ^13^C_6_, Cambridge Isotope Laboratories, Andover, MA). ApoE-containing serum-free media were collected after 48 hours. Media from ApoE2 and ApoE4 expressing cells were pooled for use as ^13^C_6_-leu enrichment standards as previously described [29].

### Cerebrospinal Fluid (CSF) and Plasma

Human CSF and plasma were collected from healthy young (22–49 years), cognitively-normal volunteers with a familial history (parent or grandparent) of AD and enrolled in studies approved by the Institutional Review Board of Washington University [31]. Participants were infused with ^13^C_6_-leu (2 mg/kg/h) for 9 hours and CSF was collected hourly for 48 h [30], [31]. Plasma was collected hourly for 14 h, followed by collection every odd hour. ApoE genotype was determined by PCR in the Washington University ADRC Genetics Core [52].

### Isolation of ApoE from CSF or Plasma and Tryptic Digestion

ApoE was isolated from 0.25 mL CSF and 1.5 mL pooled ApoE2/E4 astrocyte media using PHM-L liposorb, reduced and alkylated, and digested with trypsin as described [29]. Plasma ApoE was prepared as described for CSF with the following exceptions: ApoE was isolated from 50 µL of plasma by immunoprecipitation with WUE4, a monoclonal total ApoE antibody [53].

### Mouse^ 13^C_6_-leucine Injection, Tissue Lysis, and ApoE Isolation

All animal studies were approved by the Animal Studies Committee of Washington University School of Medicine. Homozygous PDAPP mice containing the human *APP* V717F mutation were generated on a mixed background of DBA/2J, C57BL/6J, and Swiss Webster (gift from Eli Lilly and Co.). To generate APP transgenic mouse models with human ApoE, PDAPP mice were crossed with human *APOE* knock-in mouse models in which the endogenous murine *APOE* gene is replaced with the *APOE3* or *APOE4* gene (gift from Dr. Patrick Sullivan, Duke University) [39]. 3.5 month old ApoE3/E3 and ApoE4/E4 TR mice were injected intraperitoneally with ^13^C_6_-leu (200 mg/kg) and the brain tissue was harvested and plasma collected after predetermined time points (either 20 min, 40 min, 1, 4, 7, 13, or 19 h). 3–5 mice were used for each time point. One hemisphere of cortex was then lysed by the addition of 1% triton X-100 lysis buffer [1% triton X-100, 150 mM NaCl, 50 mM Tris-HCl, 1× complete protease tablet (Roche)], followed by sonication and centrifugation to remove the tissue debris. ApoE was immunoprecipitated from the cortex using WUE4 antibody. Briefly, WUE4 antibody was coupled to Protein G Sepharose 4 Fast Flow beads (GE Lifesciences) overnight at 4°C and the bound antibody was cross-linked using dimethyl pimelimidate (20 mM). The tissue lysate was then incubated with the WUE4- coupled beads O/N at 4°C. The beads were then washed three times with PBS and three times with triethylammonium bicarbonate (25 mM). The precipitated ApoE was then eluted from the beads using formic acid, the formic acid was removed via vacuum evaporation, and the protein resuspended in 20% acetonitrile/80% triethylammonium bicarbonate. 500 ng of trypsin (Promega) was then added to the samples and the digest was carried out for 18 h at 37°C.

### Identification of MRM Transitions

See supplemental data for detailed LC/MS/MS methods (Table S3 and Table S4).

### NanoLC Tandem MS and Quantitation

#### Human studies

^13^C_6_-leu labeled and unlabeled ApoE isoform-specific peptides [**L**GADMEDVc_112_GR, **L**GADMEDVR_112_, **L**AVYQAGAR, and c_158_ **L**AVYQAGAR (“c” indicates alkylated cysteine residue M+57)] were separated by reverse phase on a nanoLC-2D-Ultra (Eksigent Technologies, Dublin, CA). Peptides were detected by a TSQ Vantage (ThermoFisher Scientific, San Jose, CA) operating in MRM mode as described in supplemental data, and quantitated using ThermoFisher’s Xcalibur® Processing setup and QuanBrowser software (version 2.0.7).

#### Mouse studies

^13^C_6_-leu labeled and unlabeled ApoE common peptide SWFEP**L**VEDMQR was detected by a Xevo TQ-S (Waters Corporation, Milford, MA) as described in supplemental data, and quantitated using Waters MassLynx 4.1 software. Human and mouse LC-MS results were exported to Microsoft Office Excel spreadsheets and GraphpadPrism5 for further statistical analyses.

### Fractional Synthesis Rate (FSR) and Fractional Clearance Rate (FCR) Calculations

Plasma ^13^C_6_-leu tracer to trace ratio (TTR) (the molar ratio of labeled to unlabeled species) was determined using GC/MS [54], and used to represent the precursor pool enrichment for ApoE synthesis. The FSR of ApoE isoforms was calculated by dividing the slope of the linear regression during the incorporation phase of ApoE (h4–16 for CSF, and h0–4 for plasma) by the average plateau of free ^13^C_6_-leu enrichment in plasma [31], [55]. For the mouse studies, FSR curves were generated using the 20 min and 40 min labeled/unlabeled ApoE values that had been normalized to plasma ^13^C_6_-leu values obtained from the 20 min time point. The ApoE isoform monoexponential slope FCR was calculated using the natural log slope for the clearance portion (h28–44 for CSF, and h10–19 for plasma) of the ApoE TTR time course [31]. For the mouse studies, the clearance portion of the curve was considered to be 1 to 13 hrs.

### Compartmental Modeling

Compartmental modeling was performed using the SAAM II program (SAAM institute, University of Washington, Seattle). The fractional turnover rate (FTR) of peripheral plasma ApoE was determined using a compartmental model (Fig. 2*A*) that features a plasma leucine precursor pool, a plasma ApoE pool that turns over, and a non-plasma exchange compartment that is required to completely account for the shape of the time course. The model used each subject’s measured plasma ^13^C_6_-leu TTR time course and accounted for isotopic dilution between plasma leucine and ApoE production. The plasma ApoE FTR is the rate constant for irreversible loss of ApoE. The model used for CNS ApoE (Fig. 4*A* *)* is based on a model routinely used for very low density lipoprotein apoB-100 turnover kinetics [56]. The model described the shape of the ApoE TTR time course as a combination of a delay and a turning over compartment. The residence time (RT) of ApoE in the whole system was calculated as the sum of the RT for the delay and turnover compartments, and the whole-system FTR was calculated as the inverse of whole-system RT.

### Statistical Analysis

CSF results were analyzed by a repeated measure analyses of variance (ANOVA) in which genotypes are between subjects and the peptides are within group factors. For the mouse studies, statistical significance between the two genotypes was calculated using analysis of covariance (ANCOVA) for the FSR and FCR slopes.
