# Correction: In Vivo Human Apolipoprotein E Isoform Fractional Turnover Rates in the CNS

**DOI:** 10.1371/annotation/9bd329b7-cdea-45ec-a4aa-a173855dc0b9

**Published:** 2012-07-03

**Authors:** Kristin R. Wildsmith, Jacob M. Basak, Bruce W. Patterson, Yuriy Pyatkivskyy, Jungsu Kim, Kevin E. Yarasheski, Jennifer X. Wang, Kwasi G. Mawuenyega, Hong Jiang, Maia Parsadanian, Hyejin Yoon, Tom Kasten, Wendy C. Sigurdson, Chengjie Xiong, Alison Goate, David M. Holtzman, Randall J. Bateman

Description: Due to errors introduced in the production process, the Supporting Information files were excluded. They can be found here:

Table S1: 

**Figure:**
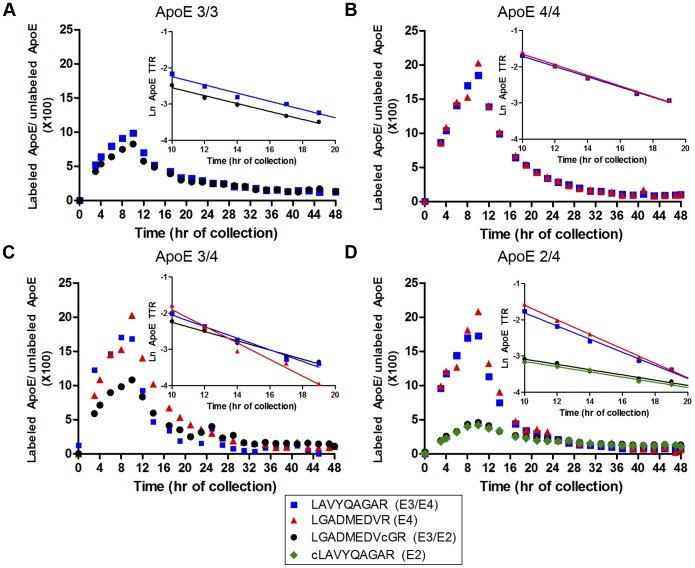


 [^]

